# Supplementary figures and images for: Differential translation of mRNA isoforms underlies oncogenic activation of cell cycle kinase Aurora A
Source: eLife. 2023 Jun 29;12:RP87253. doi: 10.7554/eLife.87253 (PMC10328522; doi:10.7554/eLife.87253)

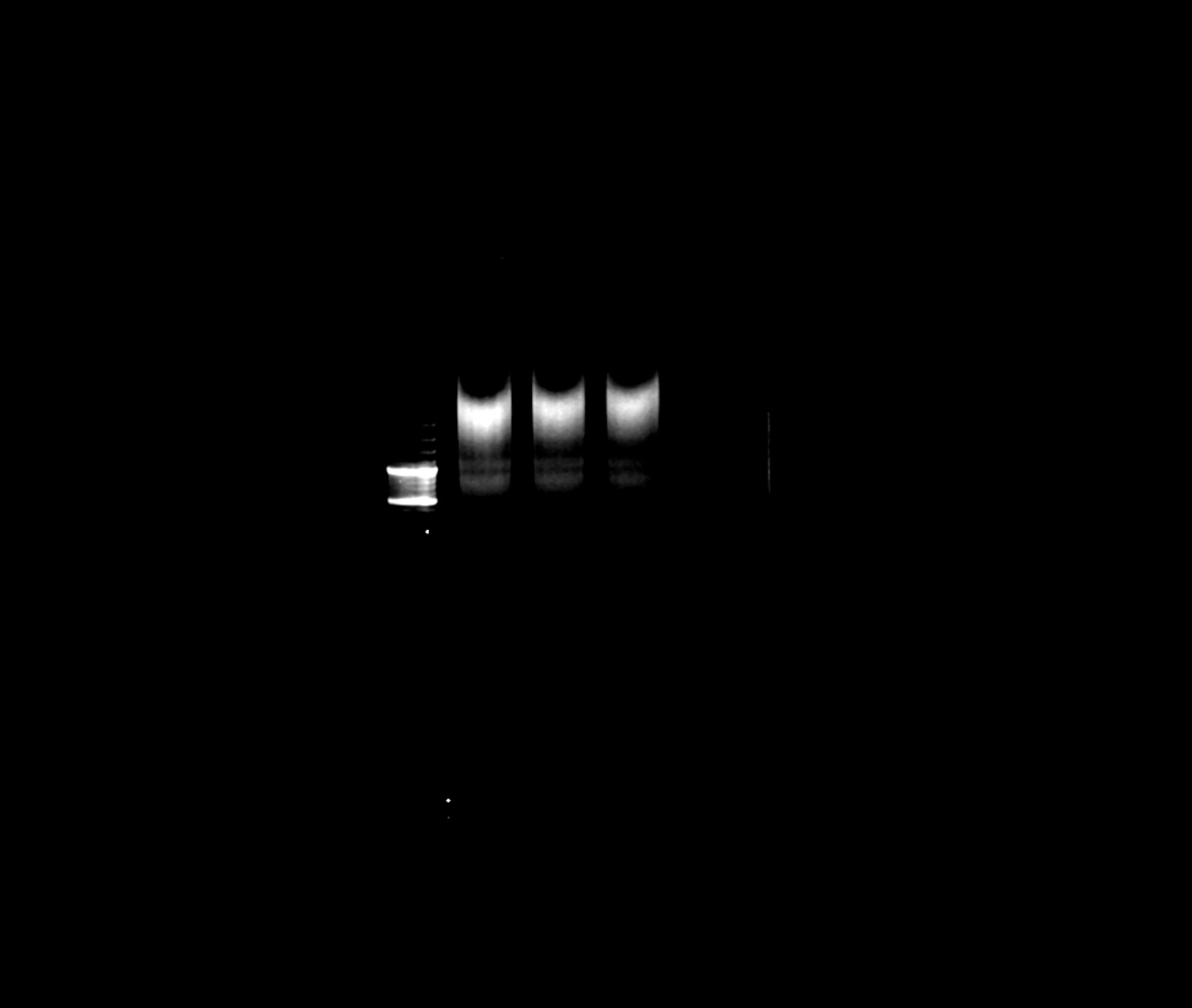

Supplement: Figure 2—source data 1. [file elife-87253-fig2-data1.zip › Figure 2-Source Data 1/3'RACE.tif]

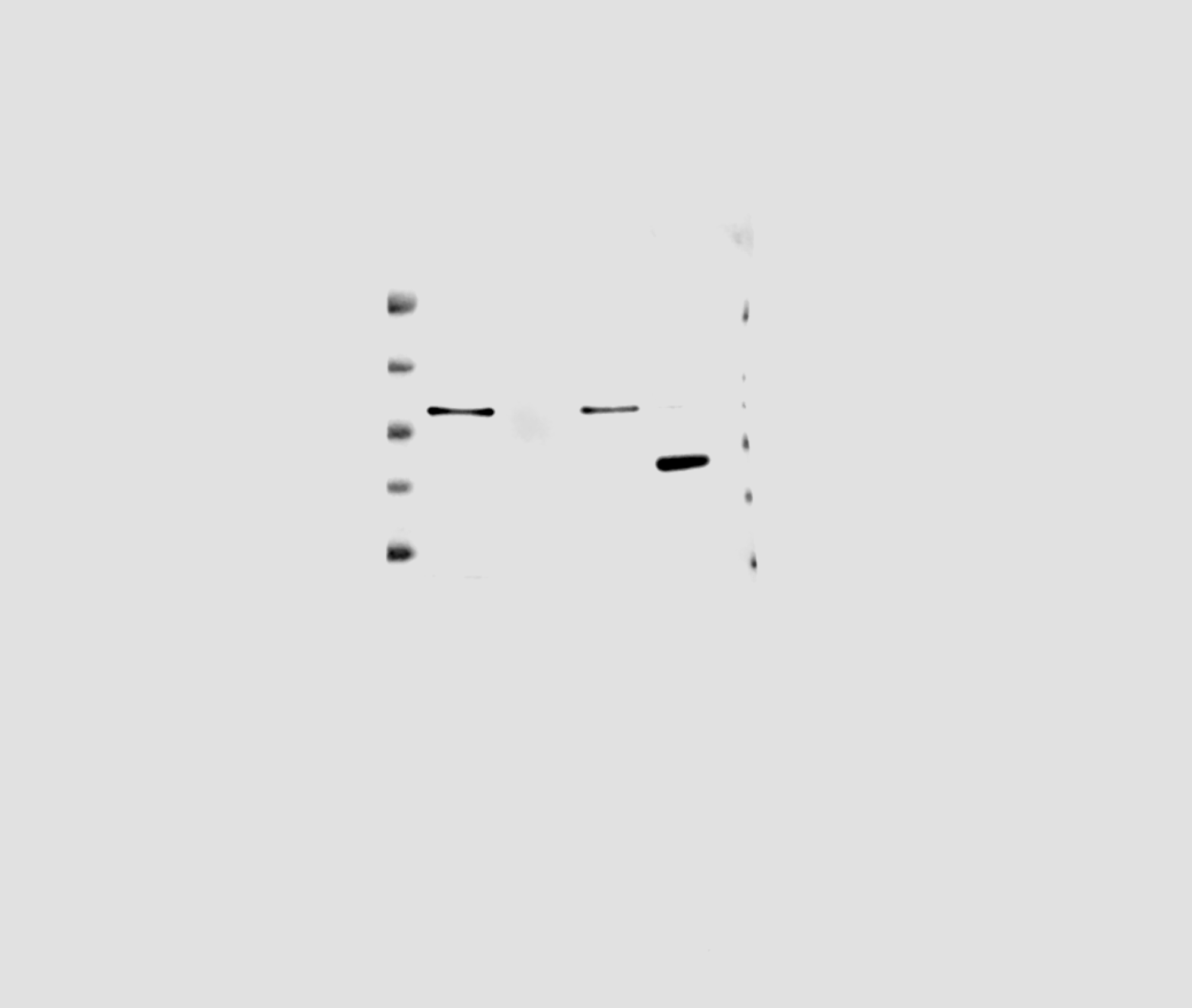

Supplement: Figure 3—source data 2. [file elife-87253-fig3-data2.zip › Figure 3-Source Data 2/Flag-Γêå_3XFlag_anti-Flag.tif]

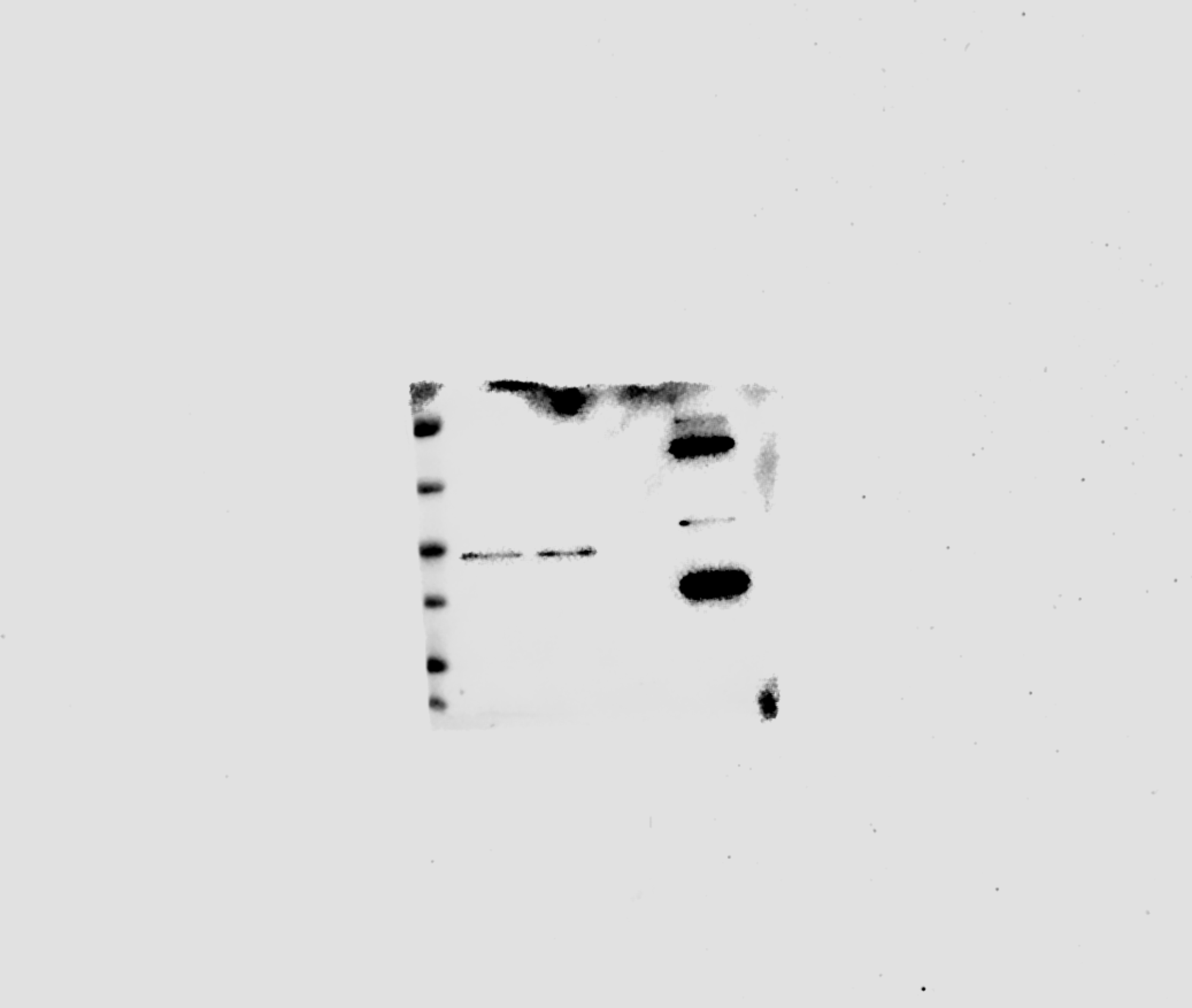

Supplement: Figure 3—source data 2. [file elife-87253-fig3-data2.zip › Figure 3-Source Data 2/Flag-Γêå_3XFlag_anti-mCherry.tif]

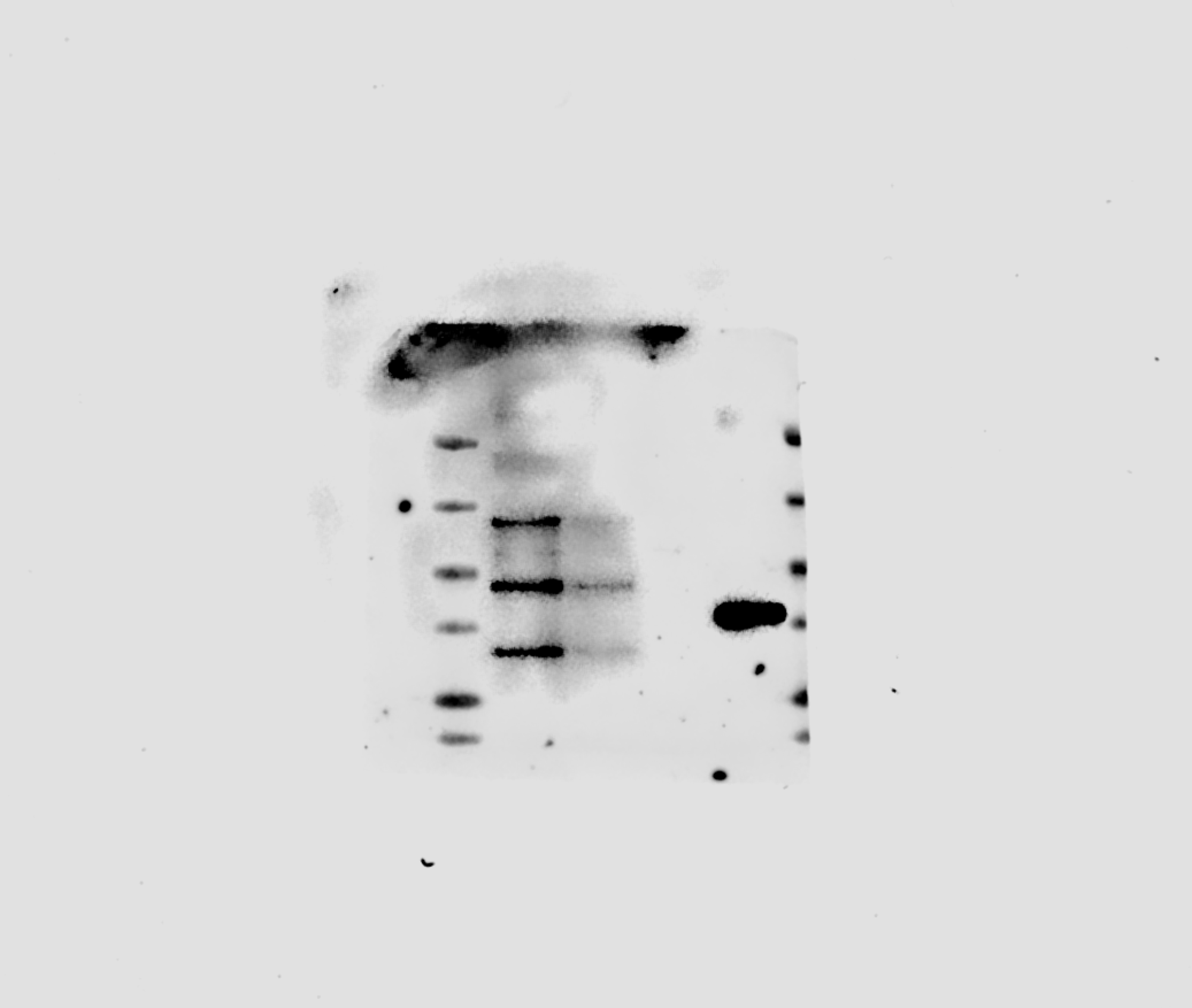

Supplement: Figure 3—source data 2. [file elife-87253-fig3-data2.zip › Figure 3-Source Data 2/Γêå_Puromycin_anti-mCherry.tif]

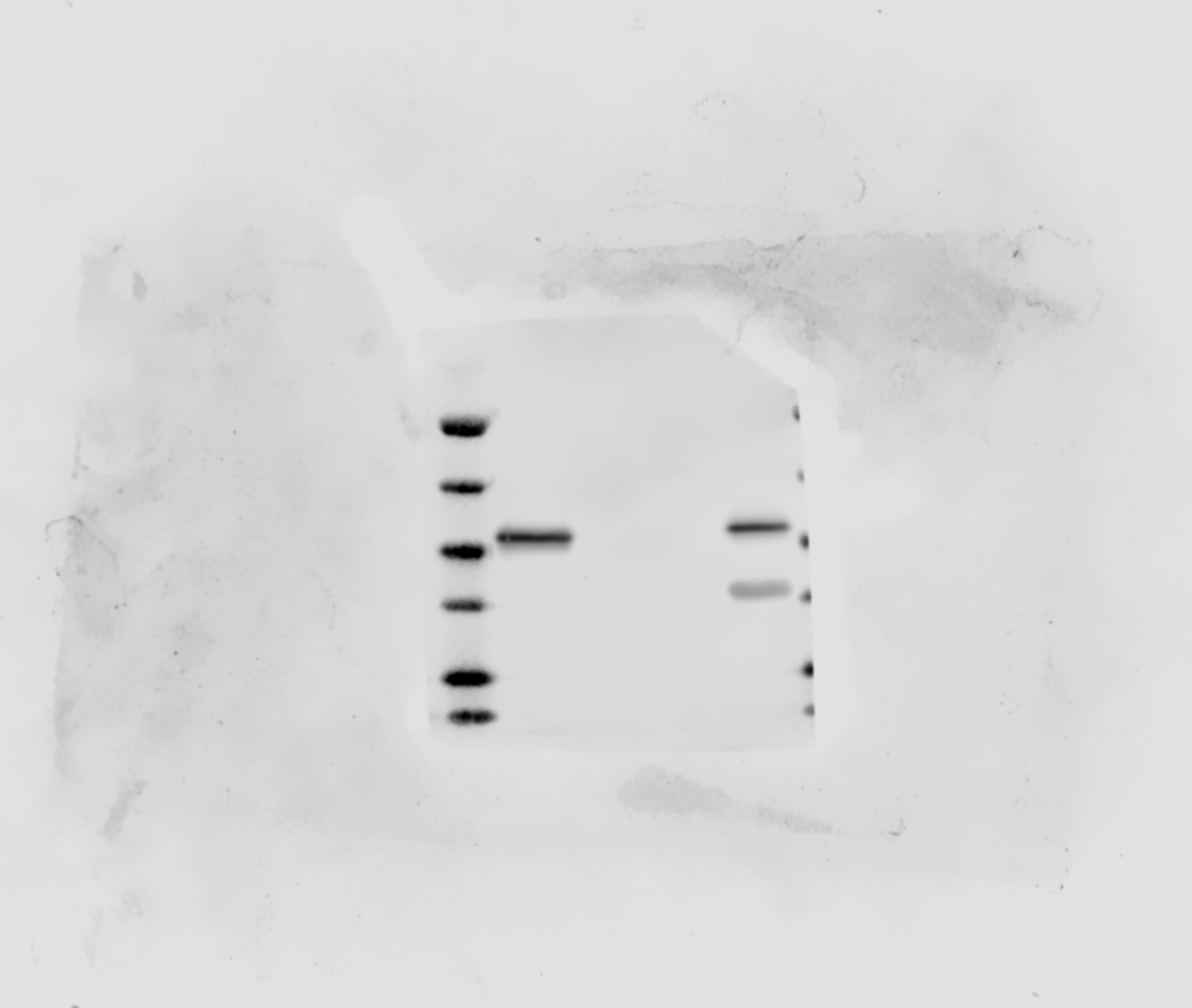

Supplement: Figure 3—source data 2. [file elife-87253-fig3-data2.zip › Figure 3-Source Data 2/Flag-Γêå_Puromycin_anti-Flag.tif]

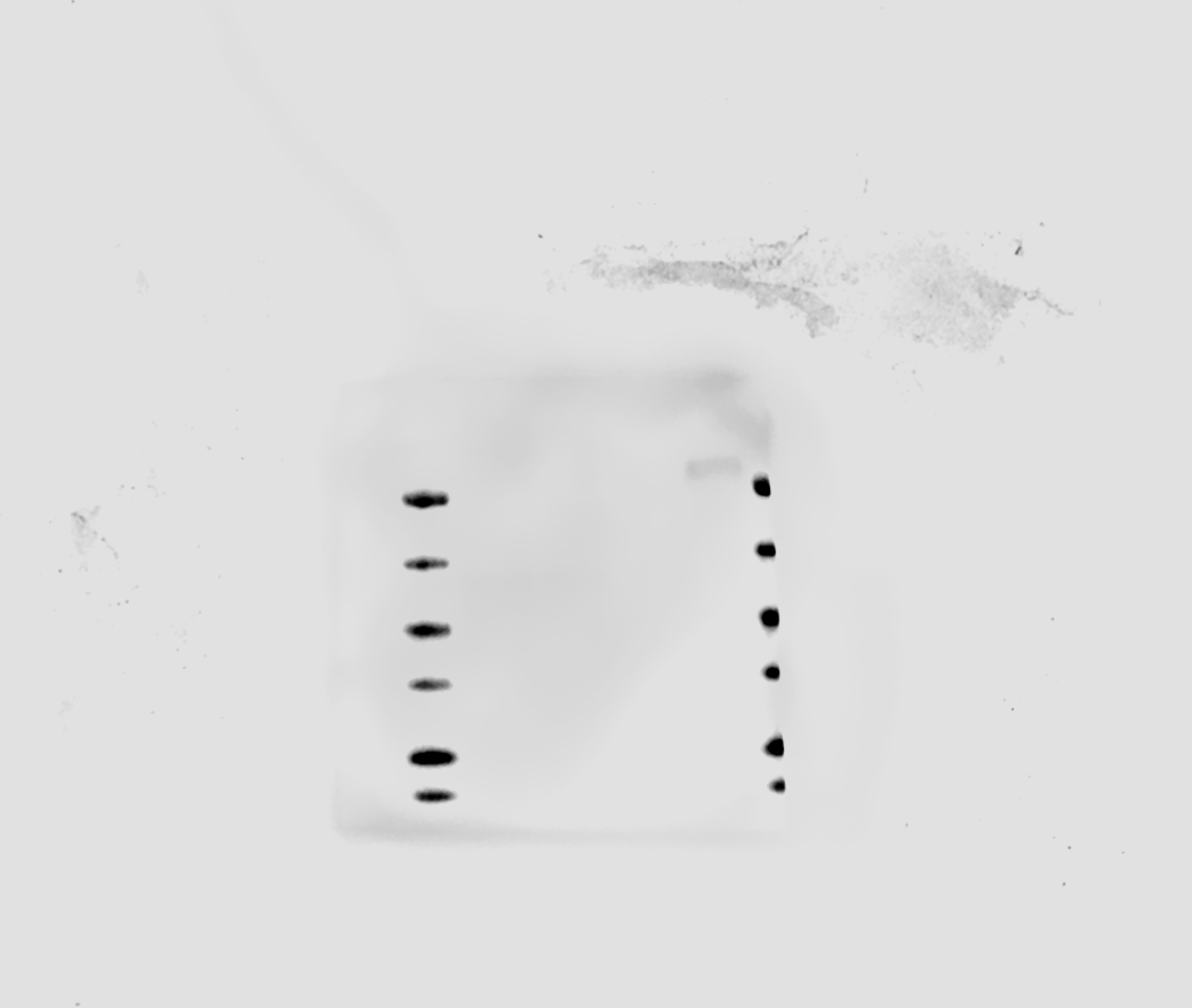

Supplement: Figure 3—source data 2. [file elife-87253-fig3-data2.zip › Figure 3-Source Data 2/Γêå_Puromycin_anti-Flag.tif]

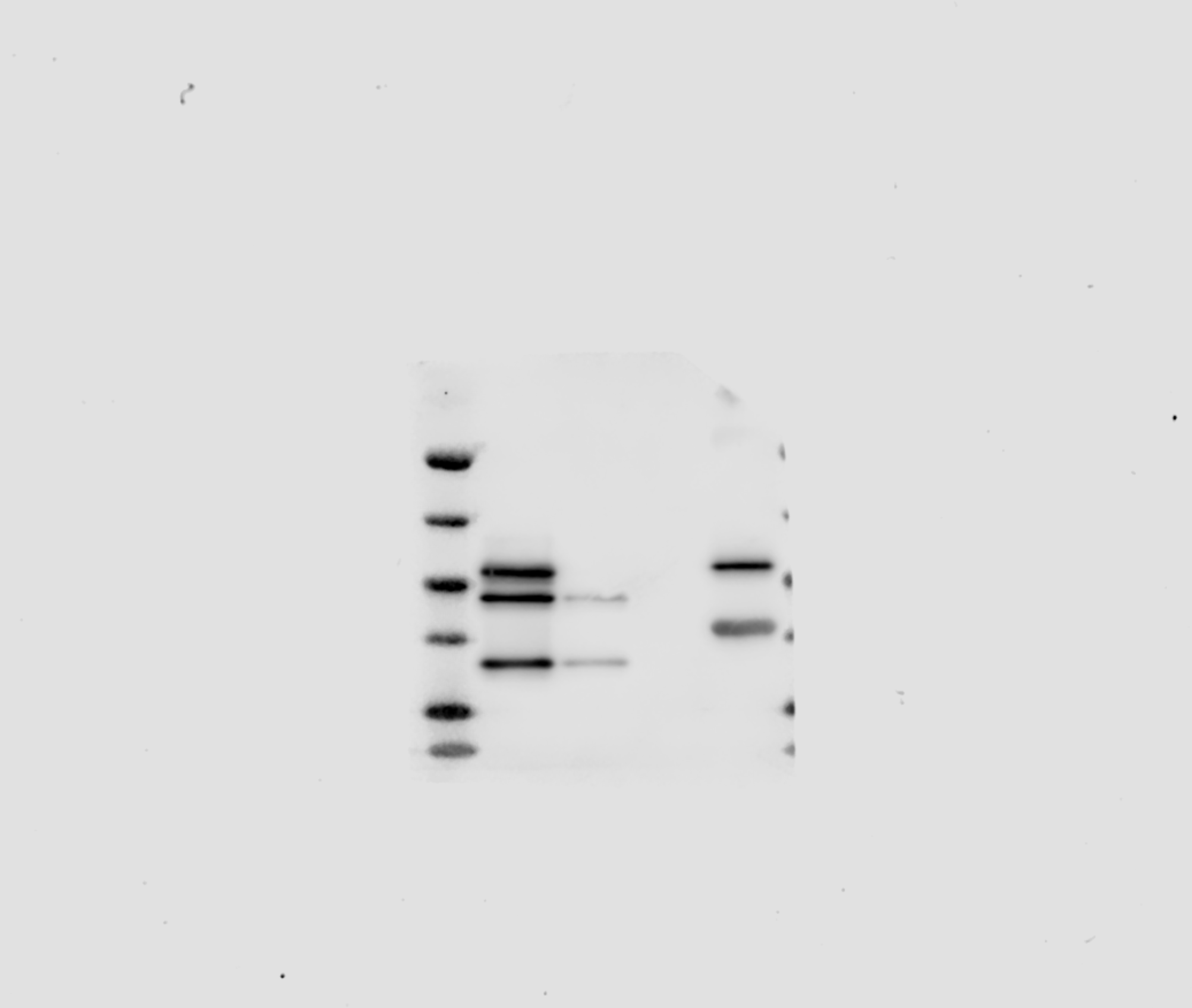

Supplement: Figure 3—source data 2. [file elife-87253-fig3-data2.zip › Figure 3-Source Data 2/Flag-Γêå_Puromycin_anti-mCherry.tif]

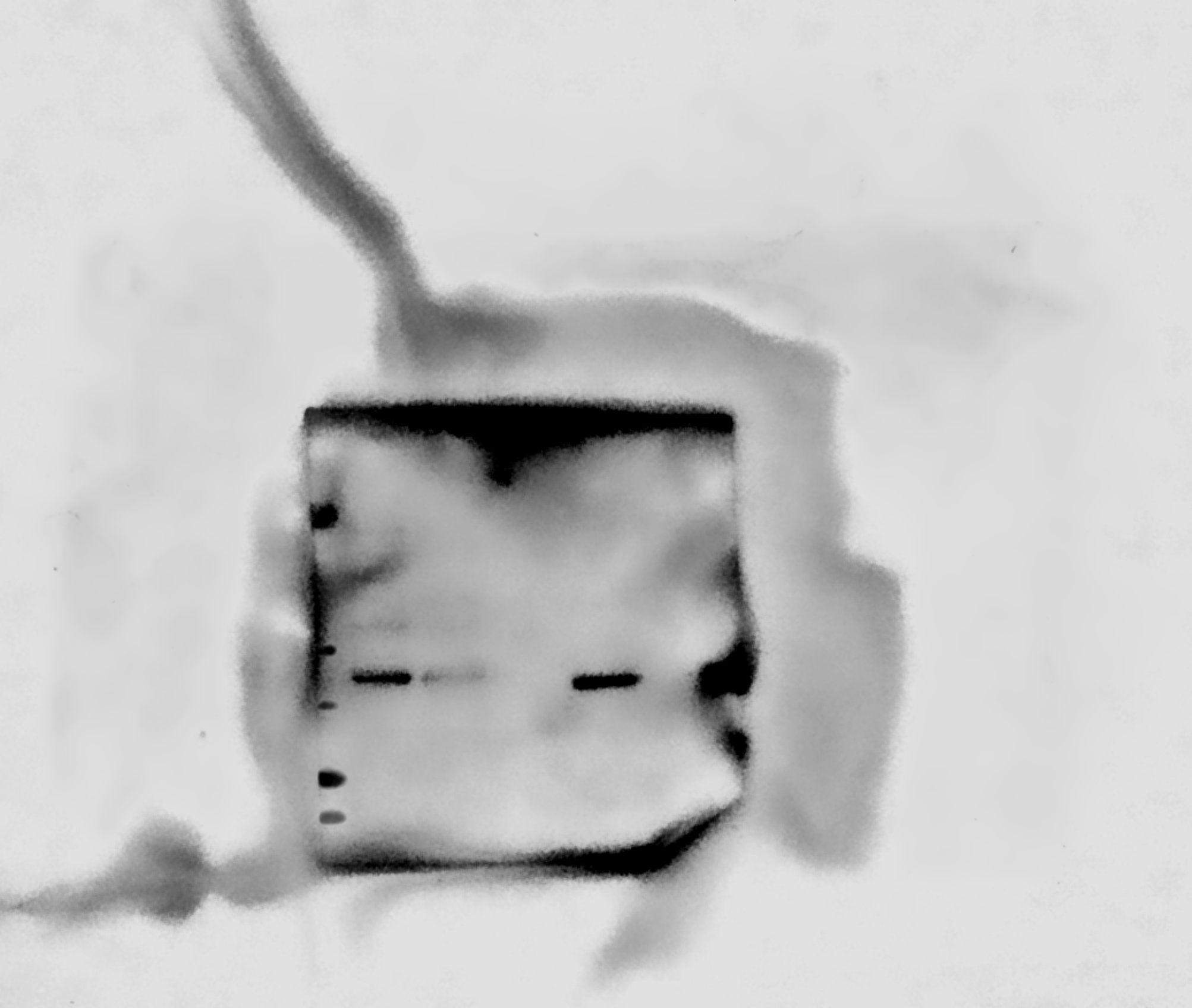

Supplement: Figure 3—source data 2. [file elife-87253-fig3-data2.zip › Figure 3-Source Data 2/Γêå_Puromycin_anti-GFP.tif]

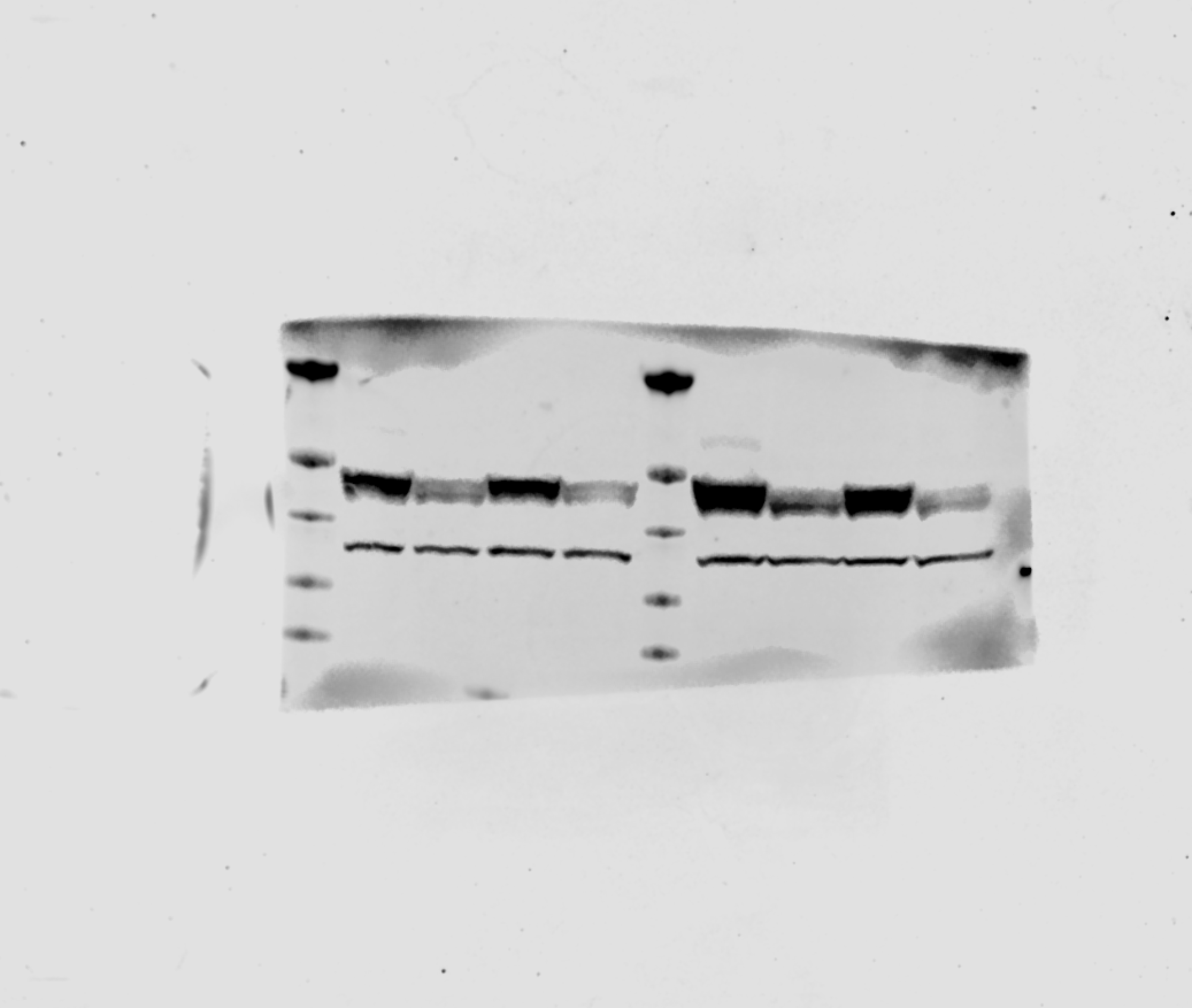

Supplement: Figure 6—source data 1. [file elife-87253-fig6-data1.zip › Figure 6-Source Data 1/WB + CHX.tif]

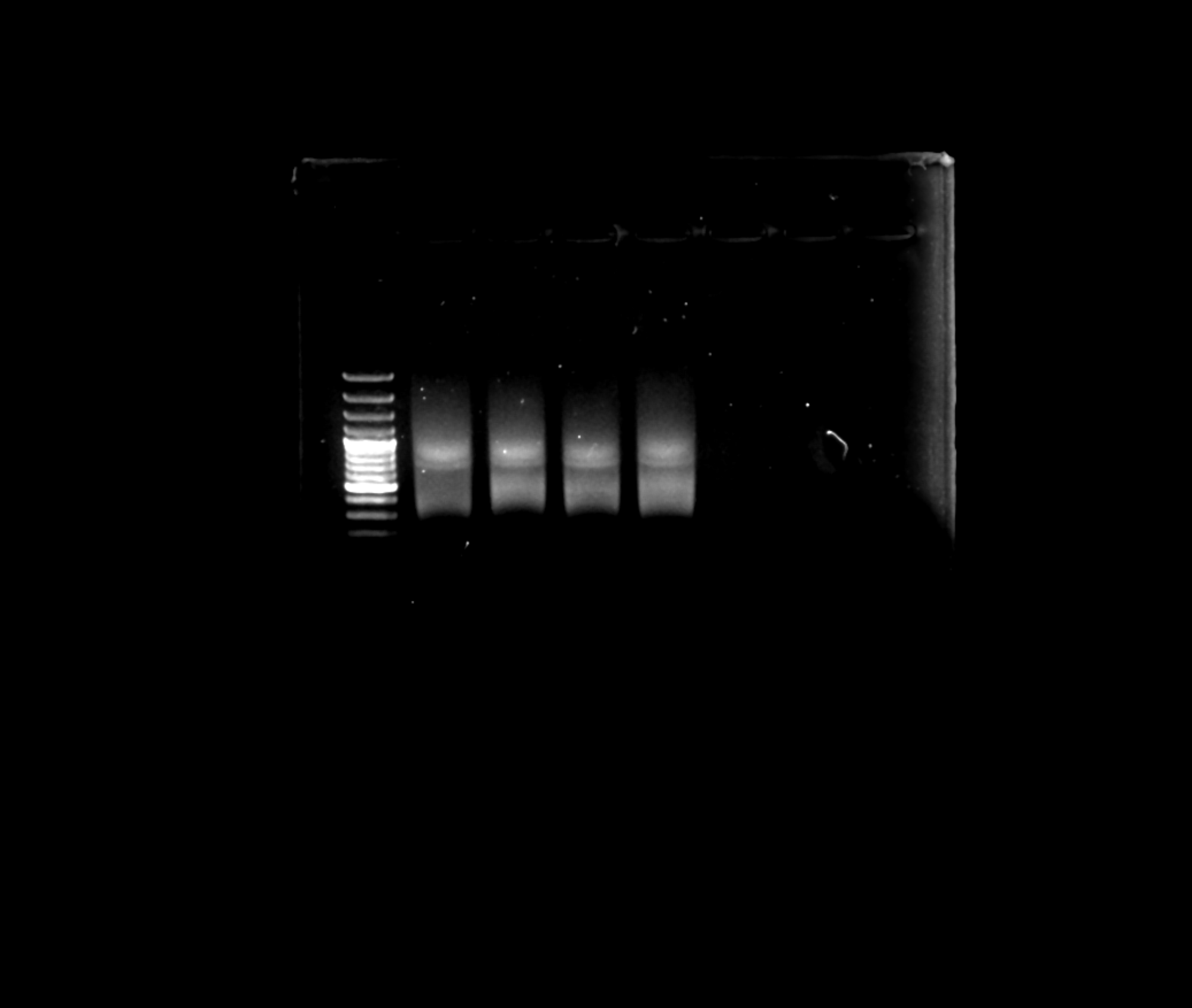

Supplement: Figure 6—source data 1. [file elife-87253-fig6-data1.zip › Figure 6-Source Data 1/3'RACE.tif]

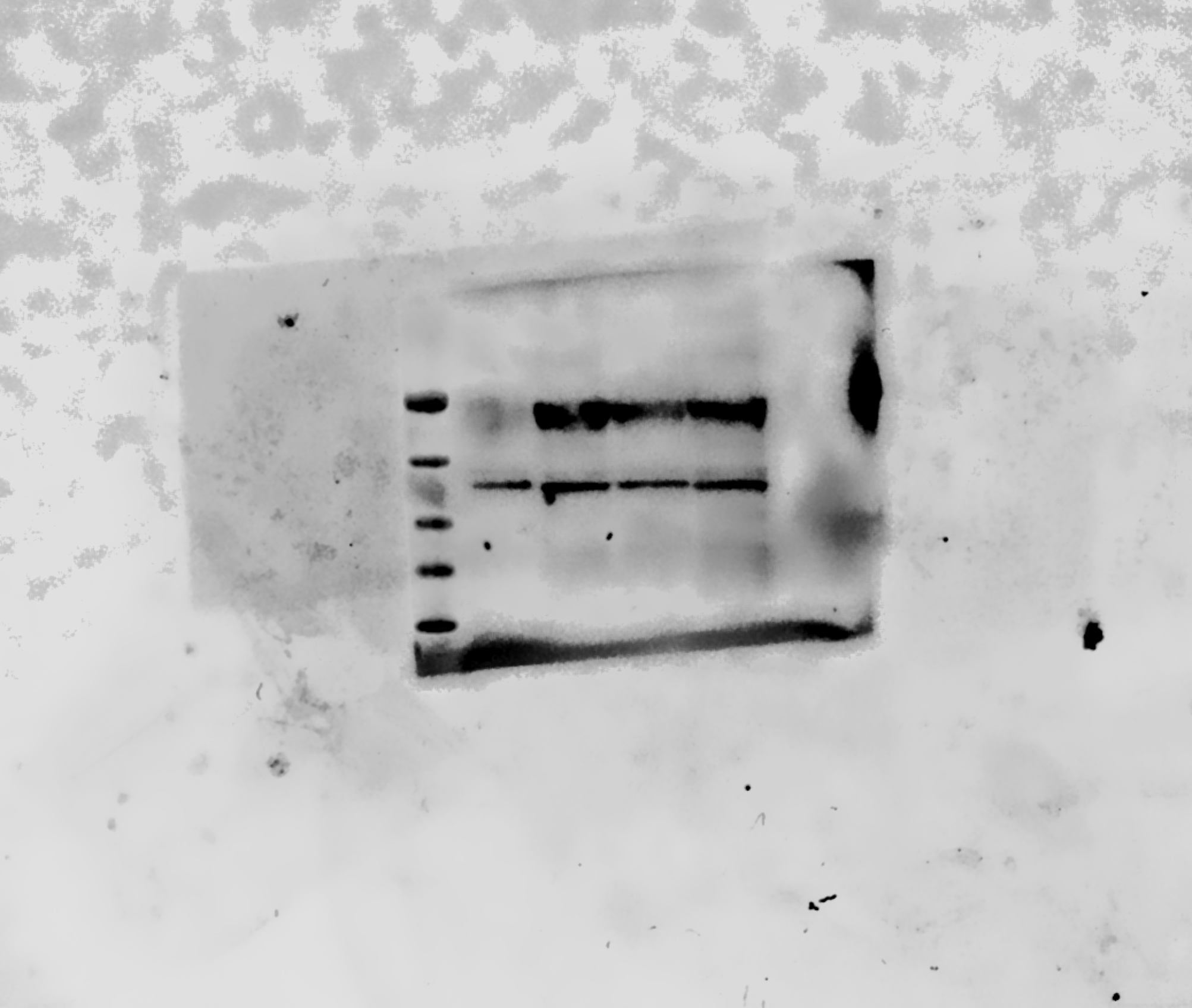

Supplement: Figure 6—source data 1. [file elife-87253-fig6-data1.zip › Figure 6-Source Data 1/WB.tif]

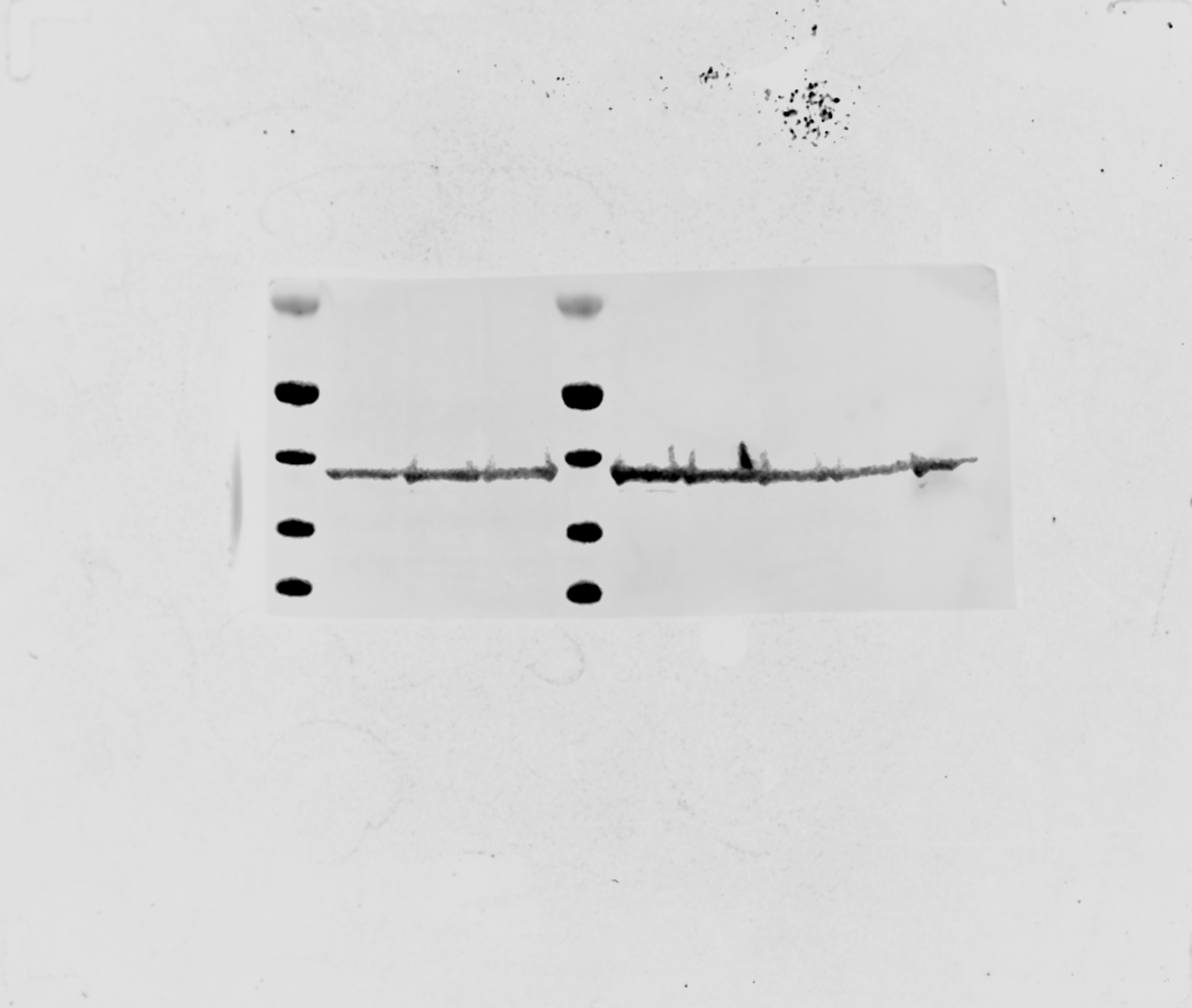

Supplement: Figure 6—figure supplement 1—source data 1. [file elife-87253-fig6-figsupp1-data1.zip › Figure 6-figure supplement 1-Source Data 1/anti-GAPDH.tif]

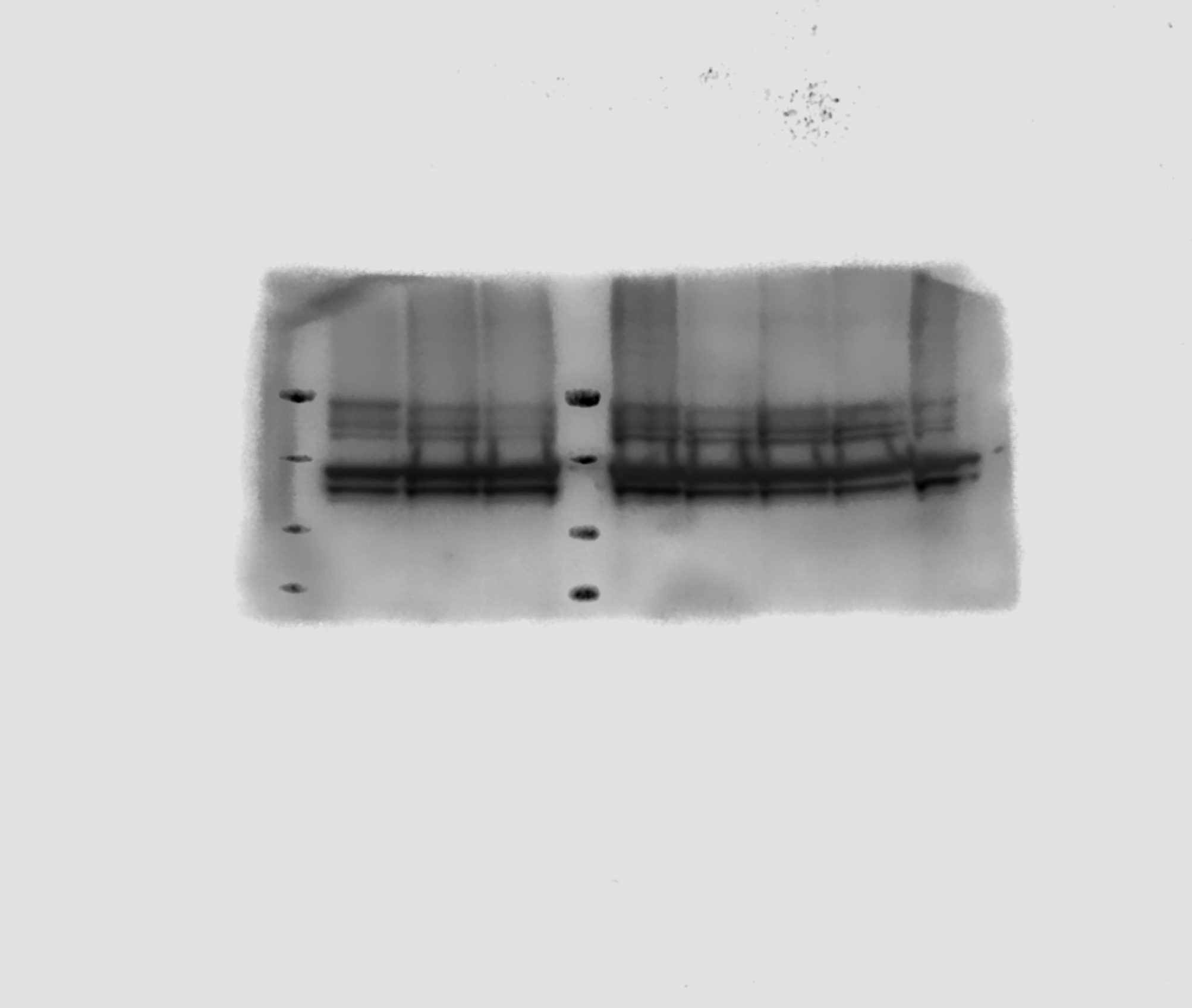

Supplement: Figure 6—figure supplement 1—source data 1. [file elife-87253-fig6-figsupp1-data1.zip › Figure 6-figure supplement 1-Source Data 1/anti-AURKA.tif]
